# Identification and Characterization of Potential Discharge Areas for Radionuclide Transport by Groundwater from a Nuclear Waste Repository in Sweden

**DOI:** 10.1007/s13280-013-0395-5

**Published:** 2013-04-26

**Authors:** Sten Berglund, Emma Bosson, Jan-Olof Selroos, Mona Sassner

**Affiliations:** 1 HydroResearch AB, Stora Marknadsvägen 15S (12th Floor), 183 34 Täby, Sweden; 2 Swedish Nuclear Fuel and Waste Management Co (SKB), Box 250, 101 24 Stockholm, Sweden; 3 DHI Sverige AB, Svartmangatan 18, 111 29 Stockholm, Sweden

**Keywords:** Contaminant transport, Hydrology, Hydrogeology, Safety assessment, Geosphere, Biosphere, Forsmark

## Abstract

This paper describes solute transport modeling carried out as a part of an assessment of the long-term radiological safety of a planned deep rock repository for spent nuclear fuel in Forsmark, Sweden. Specifically, it presents transport modeling performed to locate and describe discharge areas for groundwater potentially carrying radionuclides from the repository to the surface where man and the environment could be affected by the contamination. The modeling results show that topography to large extent determines the discharge locations. Present and future lake and wetland objects are central for the radionuclide transport and dose calculations in the safety assessment. Results of detailed transport modeling focusing on the regolith and the upper part of the rock indicate that the identification of discharge areas and objects considered in the safety assessment is robust in the sense that it does not change when a more detailed model representation is used.

**Electronic supplementary material:**

The online version of this article (doi:10.1007/s13280-013-0395-5) contains supplementary material, which is available to authorized users.

## Introduction

In many environmental applications involving risk assessment of subsurface contaminants, the analysis must consider the transport of potentially harmful substances from source locations to places where consequences for man and the environment might arise and need to be quantified. Transport modeling is therefore often needed in these applications, in order to determine (i) where exposure to the contaminants could take place, and who or what would be exposed; (ii) transport times and when the contaminants reach the identified receptors; and (iii) the amounts of contaminants transported and the resulting concentrations and mass fluxes where exposure could take place. Different types of models are needed for different modeling purposes. For example, distributed groundwater flow models are useful for analyzing transport paths and to identify discharge areas where contaminants reach surface ecosystems, whereas transport models that consider various biogeochemical reactions might be needed to assess the consequences of the contamination.

Performance and safety assessments of geological repositories for nuclear waste constitute important applications for the methods and models outlined above. The Swedish Nuclear Fuel and Waste Management Company (SKB) recently performed an assessment of the long-term radiological safety of a deep geological repository for spent nuclear fuel at the Forsmark site (SKB 2011; Kautsky et al. 2013). In this safety assessment, SKB used a suite of transport models to quantify radionuclide transport through the engineered and geological barriers and in the biosphere. Some of these models were integrated in a ‘model chain’ and used directly in the dose and risk calculations (Selroos and Painter 2012), whereas others were utilized in supporting modeling activities intended to produce input data or test assumptions made in model development. An overview of flow and transport models used in SKB safety assessments is given by Berglund et al. (2009).

In the SKB safety assessment of the spent fuel repository, radionuclide transport and dose modeling of the biosphere reported by Avila et al. (2010, 2013) were used as a basis for quantifying the radiological consequences of hypothetical future releases from the repository (SKB 2010, 2011; Kautsky et al. 2013). This biosphere modeling was based on a landscape model consisting of interconnected ‘biosphere objects’, e.g., lakes and associated catchment areas where radionuclide-contaminated groundwater could discharge and affect the biosphere in the event of a release from the repository (Lindborg 2010; Berglund et al. 2013). For these biosphere objects, radionuclide transport and doses were calculated using compartment models (Avila et al. 2013). The identification of discharge areas and biosphere objects, and the description of the processes governing solute transport to and within them are examples of important aspects of the biosphere analyses, and hence of the overall dose and risk assessment, that were investigated in supporting modeling activities.

This paper describes some of the transport modeling that was carried out in order to support the biosphere analyses in the safety assessment. Specifically, it presents and discusses the relatively large-scale solute transport modeling performed in order to locate the discharge areas used as biosphere objects and the more detailed modeling of transport in the near-surface system (the upper part of the bedrock and the overlying regolith) intended to analyze detailed discharge patterns and solute spreading. The modeling discussed in this paper consists of particle tracking and advection–dispersion simulations, which means that it is restricted to non-reactive transport. Modeling that takes processes acting to retain and/or transform radionuclide transport has been reported elsewhere (see, e.g., Grandia et al. 2011; Avila et al. 2013; Piqué et al. 2013).

Our main objectives are to describe and evaluate an integrated modeling approach for identification and characterization of biosphere objects, and to investigate uncertainties in the modeled discharge locations. In a wider perspective, the analysis demonstrates a methodology where flow and transport modeling based on data from extensive site investigations is used to connect potential subsurface sources with the surface ecosystems for which the consequences of the waterborne contamination are assessed. Although the presentation considers a specific application and site, this methodology is believed to be useful for a wider class of environmental applications involving contaminants residing in bedrock or at some depth in unconsolidated deposits.

The overall context and purpose of the work presented herein are given by the safety assessment of the planned nuclear waste repository; see SKB (2010, 2011) for descriptions of the development of assessment and modeling approaches in this field. The scientific background of the specific modeling activities presented in this paper is given by recent developments within the modeling of flow and transport in fractured rock, regolith, and surface water systems. Concerning flow and transport in fractured rock, research performed in the context of nuclear waste disposal provides the main scientific input to the present study (see Hodgkinson et al. 2009 for an overview of a related research program). The development of flow path-based transport models is an important aspect of this research, see, e.g., Cvetkovic and Frampton (2012); a model application using data from the Forsmark site is reported by Cvetkovic and Cheng (2011), whereas Selroos and Painter (2012) present an analysis of transport modeling results from SKB’s safety assessment.

For the modeling of surface hydrology and near-surface hydrogeology, scientific background and support is provided by a number of research studies that use data and models from Forsmark to address general (i.e., not necessarily related to nuclear waste) hydrological and solute transport issues (e.g., Jarsjö et al. 2008; Juston et al. 2009; Destouni et al. 2010; Persson et al. 2011). Furthermore, model studies of present and future hydrological conditions at Forsmark have recently been presented by Bosson et al. (2012a, b); these studies are based on the same conceptual and numerical models as the present work.

## Materials and Methods

### Site Overview

In June 2009, Forsmark in Eastern Sweden was selected by SKB as the site for the planned final repository for spent nuclear fuel, which in the SKB concept is located at a depth of ca. 500 m in the bedrock (Kautsky et al. 2013; Lindborg et al. 2013). This shoreline area is characterized by a small-scale topography at low altitude. The whole area is located below the highest coastline associated with the last glaciation, and large parts of it emerged from the Baltic Sea only during the last 2000 years.

The flat topography and the still ongoing shore-level regression of ca. 6 mm per year strongly influence the current landscape (SKB 2008). In particular, the combined effect of land uplift and a flat topography is a fast shoreline displacement that has resulted in a very young terrestrial system containing a number of newborn shallow lakes and wetlands. Sea bottom is continuously transformed into new terrestrial areas or freshwater lakes, and lakes and wetlands are successively covered by peat. The lakes themselves are also of a specific type that is found only in Northern Uppland. Shallow and with sediments rich in calcium, the lakes are unique in Sweden. Till is the dominant Quaternary deposit, and hence the main component of the regolith overlying the bedrock, whereas granite is the dominant rock type.

According to the average annual water balance of the site investigation area, as estimated from long-term regional observations and confirmed by local measurements and modeling during the site investigations, the annual precipitation, evapotranspiration, and runoff are approximately 560, 400, and 160 mm, respectively (Johansson 2008). The hydrogeological conditions in the area above the planned repository are characterized by a shallow groundwater table that follows the topography in the regolith, a set of relatively highly transmissive horizontal structures, often referred to as sheet joints, in the upper ca. 150 m of the bedrock, and very few fractures, which have relatively low transmissivities, at larger depths in the rock (Selroos and Follin 2010; Berglund et al. 2013).

In the radionuclide transport and dose modeling of Forsmark, areas with lakes or streams surrounded by wetlands are of special interest. In particular, these areas are important when defining and describing transport conditions in the biosphere objects constituting the main components of the landscape model. This is because they are discharge areas for groundwater, and therefore some of them constitute potential future discharge areas for radionuclide-contaminated groundwater from the repository. The landscape model consists of a set of interconnected biosphere objects. Due to shoreline displacement and other processes contributing to formation, infilling and terrestrialization of lakes and wetlands, these objects are subjected to a succession. After an initial period of submerged (sea-covered) conditions, this succession often includes lake, wetland, and terrestrial stages. In particular, lakes will gradually decrease in size and become wetlands, and then possibly further develop into land areas that, in some cases, may be suitable for agriculture (Lindborg et al. 2013). This succession changes the conditions for land use and potential exposure to contaminants, and is therefore an important component of the landscape and radionuclide transport models developed within the safety assessment (Avila et al. 2010; Lindborg 2010; SKB 2010).

### Conceptualization and Modeling Methodology

**Fig. 1:**
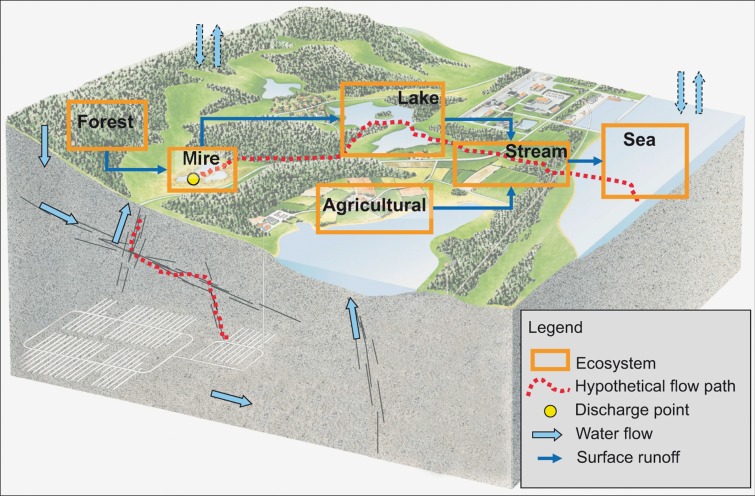
Conceptual model of solute transport along a hypothetical flow path (*dotted line*) from the spent fuel repository (indicated by the *white lines* in the *gray* bedrock part of the model) up to a discharge point (the *larger dot* in the ‘Mire’ box), and further through different types of ecosystems to the sea. From Lindborg (2010)

Figure 1 shows a generic conceptual model of radionuclide transport from the planned spent fuel repository, up to the surface and further within and between different types of surface ecosystems. A hypothetical flow path, along which dissolved radionuclides could be transported, is shown as a dotted line in the figure. Note that the figure emphasizes the mainly horizontal transport near and on the surface, whereas the mainly vertical transport through fractures and deformation zones in the bedrock here is shown in greatly simplified form. This conceptual model provides a common basis for the development of site-specific hydrological, ecosystem and landscape models, which, in turn, constitute an important input when developing the models used in transport and dose calculations.

Site-specific geological and hydrological data and models are of great importance when developing conceptual and numerical transport models for a particular site. Figure 1 indicates that the transport problem at hand involves different types of systems or domains, e.g., bedrock, regolith, and surface-water systems, which require different types of data and models. The overall safety assessment, and hence also the hydrological modeling, divides the life span of the repository into different time periods characterized by different operational and/or climate conditions (Selroos and Follin 2010; SKB 2011; Näslund et al. 2013). This paper describes hydrological modeling of the initial period of temperate climate (i.e., similar to the present) after closure of the repository.

For several of the disciplines involved in the Forsmark site descriptive modeling and the associated safety assessment, primarily geology, hydrology, and hydrogeochemistry, a distinction was made between the surface system and the bedrock system. In the modeling of water flow and waterborne transport discussed herein, different numerical modeling tools were used in the development of surface system and bedrock system models. Figure S1 (in Electronic Supplementary Material) shows the division of the hydrologic cycle into different model domains and also indicates the modeling tools that have been used for each domain, i.e., ConnectFlow in the modeling focusing on the bedrock system and MIKE SHE in the surface system modeling. Descriptions of the bedrock/ConnectFlow and surface/MIKE SHE modeling activities within the safety assessment are given by Joyce et al. (2010) and Bosson et al. (2010), respectively; the reader is referred to these reports for detailed information on the modeling procedures and the numerical models.

The ConnectFlow model has its bottom boundary at a depth of ca. 1200 m, whereas the bottom boundary of the MIKE SHE models is at a depth of ca. 600 m below ground. Thus, a relatively large depth interval in the bedrock is included in both models. Furthermore, both models include a representation of the regolith, although with different degrees of detail. This means that the differences between the two modeling activities concern the purposes of the modeling and which properties and processes that are handled in detail, more than the actual model domains considered in each activity. For example, a detailed representation of the repository and the surrounding fractured bedrock is included in the ConnectFlow model, whereas the MIKE SHE model includes a detailed representation of the regolith and quantifies the hydrological processes at the surface, including the surface water system, the unsaturated zone, and exchanges with the atmosphere. In the following, the ConnectFlow and MIKE SHE models are referred to as bedrock and surface models, respectively.

The bedrock models were used to calculate flow paths from the repository to the surface. The discharge locations obtained from these flow paths were then used as a basis for the development of landscape models (Lindborg 2010) and biosphere transport and dose models (Avila et al. 2013); essentially, the discharge locations showed where contaminated groundwater could enter the biosphere, and hence which objects and areas that needed to be included in the biosphere modeling. The hydrogeological models used to calculate flow paths from the repository to discharge locations on the surface involve large model volumes and are by necessity simplified in terms of the representation of, e.g., the details of the uppermost part of the system. Therefore, more detailed hydrological models focusing on the processes in the upper bedrock and the regolith as well as the interactions between soil, vegetation, and atmosphere were also developed (Berglund et al. 2013, Bosson et al. 2010). The main purpose of these models was to provide input, such as water fluxes between different model compartments, to the biosphere transport and dose modeling.

The bedrock and surface models were produced by different modeling teams using different modeling tools, implying a need for interactions and model integration. One important aspect of model integration was the use of a common, quality-assured dataset. Furthermore, model results in terms of groundwater fluxes in the bedrock were compared and found to be in agreement (Selroos and Follin 2010). The surface modeling produced a parameterized model of the regolith, which was delivered and used in simplified form in the bedrock model. Also the upper (flux) boundary condition in the bedrock model was based on surface modeling results. Similarly, the bedrock part of the surface model was obtained from the bedrock modeling.

### Identification of Discharge Areas

In the bedrock modeling with ConnectFlow, groundwater flow paths from each deposition hole for spent fuel canisters in the repository (in total, 6916 deposition holes) to the surface were calculated. The approach taken was to track particles moving with the advective flow velocity from release points around the deposition holes until they reached the modeled ground surface. Flow paths and associated discharge points of particles (i.e., the points where the particles reach the groundwater surface) were computed in a modeling sequence where a transient continuum model, in which shoreline displacement was taken into account, was used to generate flow fields to be used in steady-state simulations with more refined, discrete representations of the fracture network in the vicinity of the repository (Joyce et al. 2010; Selroos and Painter 2012).

The particle tracking simulations providing discharge points for the biosphere landscape modeling and transport parameters for the geosphere radionuclide transport modeling were performed in some of these steady flow fields representing selected times during the period considered in the transient modeling with the bedrock hydrogeology models. This means that particles were released and traced in fixed flow fields extracted at different times during site development. In the underlying transient modeling, the shoreline was moved over a fixed surface relief corresponding to the present topography and bathymetry. Landscape development, such as infilling of lakes, was not considered in the bedrock modeling. The effects of this simplification were tested by comparing with the surface (MIKE SHE) modeling, where this effect was taken into account.

The bedrock model results discussed in the present work were obtained by particle tracking in flow fields representing every 1000 years from 0 ad to 12 000 ad. Thus, when referring to a particular set of discharge points using a specific time, this means that the particles were released simultaneously at all deposition hole positions at that time and then traced in the flow field existing at the release time. The reason for including release times before present (i.e., 0 ad and 1000 ad) was that a whole series of glacial cycles, assumed similar to the present one (Näslund et al. 2013), was considered in the safety assessment. Therefore, future periods of submerged conditions needed to be included in the modeling, and they were represented by the most recent period when the sea covered Forsmark.

### Analysis of Near-Surface Transport Conditions

The surface system modeling of hydrology and associated non-reactive transport was performed using the MIKE SHE tool (Graham and Butts 2005; Butts and Graham 2008). This modeling is reported by Bosson et al. (2010), and summarized in Berglund et al. (2013). MIKE SHE model applications based on Forsmark data have also been presented by Bosson et al. (2012a, b). The MIKE SHE modeling of surface hydrology and near-surface hydrogeology was used to support the development of the radionuclide transport model by providing model results that were transformed to water fluxes between different compartments in the transport model. The transport calculations performed with MIKE SHE included particle tracking, where flow paths are traced by particles following the flowing groundwater, and advection–dispersion simulations. In the advection–dispersion model, solutes are transported both by the modeled groundwater flow field and by dispersion, which essentially is a lumped representation of small-scale velocity variations and diffusion.

The MIKE SHE transport modeling presented here considered the conditions at 10 000 ad, and was based on a flow model developed using the modeled shoreline position and regolith and vegetation distributions representing that time. This underlying flow model was transient and used meteorological and hydrological data with a resolution of 1 day for a 1-year period as input. These input data were based on present-day site data from Forsmark. In the flow modeling, the 1-year period was repeated until stable conditions were achieved (i.e., stable, but varying during the year). The resulting transient flow field was then used in the particle tracking and advection–dispersion simulations, where it was cycled to obtain the desired simulation periods. Particle starting positions and the sources in the advection–dispersion models were placed at ca. −40 m (elevation relative to the present sea level) along flow paths obtained from the bedrock modeling in the safety assessment. This means that the surface system modeling considered transport in the uppermost part of the rock and in the regolith.

The surface modeling with MIKE SHE was made with models termed ‘local models’ (Bosson et al. 2010); these models have a relatively high horizontal resolution (20 m by 20 m) and were developed with the intention of performing detailed transport simulations of selected biosphere objects. The 10 000 ad distributions of regolith and vegetation were used, which means that processes leading to terrestrialization of present and future lakes were implicitly taken into account. At 10 000 ad all lakes within the considered local model areas have turned into terrestrial areas and the surface water system consists of a stream network only. The local model areas constitute subareas within a larger, ‘regional’ model area of lower spatial resolution (80 m by 80 m horizontally), which, among other things, was used to generate boundary conditions for the local models.

## Results

### Identification of Discharge Areas

**Fig. 2:**
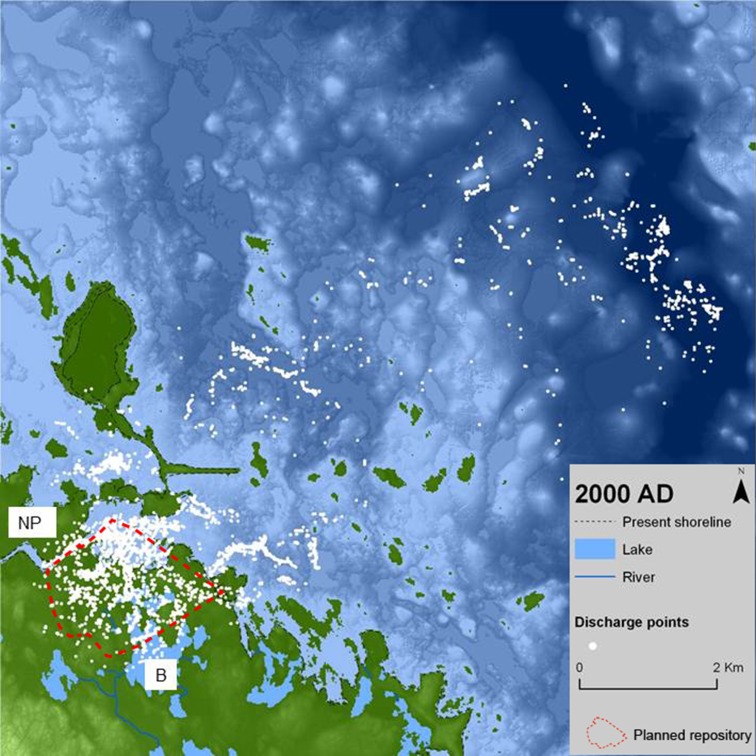
Calculated discharge points for all release times (0 ad to 12 000 ad) on a map showing the present topography and bathymetry (as indicated by ‘2000 ad’); ‘NP’ marks the location of the Forsmark nuclear power plant, ‘B’ Lake Bolundsfjärden, and the *red-dotted line* the extent of the planned repository. The topography is indicated by *different green shades* and the sea bathymetry by *blue shades*; *darker blue* or *green shades* correspond to lower elevations. After Lindborg (2010)

Figure 2 shows discharge points for all release points (in the repository) and all release times, on a map of the present topography and bathymetry. This means that discharge points are shown on a map that does not represent the site conditions when most of the particles are released, or when they reach the surface. Although perhaps slightly confusing, this presentation is made to show the overall coupling between discharge points and various features or objects in the present Forsmark landscape. As described in some detail by Lindborg (2010) this type of mapping of all discharge points is the basis for the identification of basins and biosphere objects that constitutes the starting point for the development of the biosphere landscape model.

The discharge points are to a large extent concentrated in areas near the present coastline, especially the bays just outside the nuclear power plant (Fig. 2). However, clusters of particles can be observed also at larger distances from the present coast and the discharge points are concentrated in areas with deeper water, i.e., in depressions in the bathymetry. Where the particle density in the figure is sufficiently low for patterns to be identified, there seems to be a tendency for the discharge points to form patches or clusters or to appear along lines associated with structures in the bathymetry. According to the more detailed presentation by Joyce et al. (2010), the discharge points associated with earlier release times (from 0 ad to 2000 ad) are located onshore near the planned repository, whereas the near-future discharge points (from 3000 ad to 5000 ad) follow the retreating shoreline, and many of the far-future points (from 6000 ad to 12 000 ad) are found far to the north-east.

As a part of the landscape modeling, discharge points were also displayed on maps showing the land use at different times during the considered modeling period. When producing land use maps, the modeler must make far reaching assumptions regarding the human utilization of the land (Lindborg et al. 2013). In this case, the maps are based on the assumption that all potentially arable land is used for agricultural purposes. Thus, whenever the succession of a lake to a terrestrial area results in land that can be used for agricultural production, according to the criteria used in the modeling, it is assumed to be used accordingly.

**Fig. 3:**
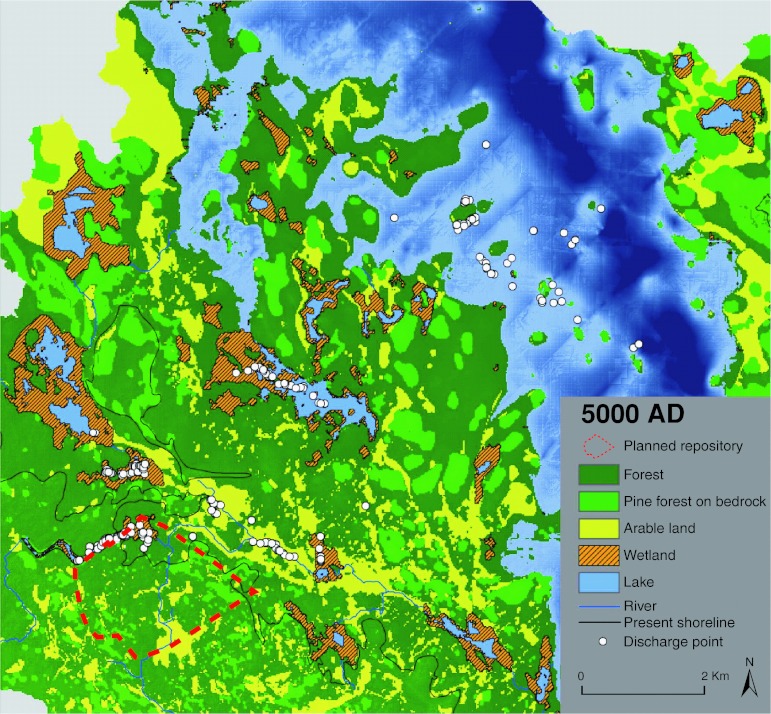
Discharge points for the 5000 ad release on a map showing land use at 5000 ad; the *red-dotted line* indicates the extent of the planned repository. The land use map is produced assuming that all potentially arable land is used for agriculture. Processes leading to infilling of lakes are taken into account. From Lindborg (2010)

Due to the shoreline displacement and the ongoing succession of lakes to wetlands and then—under certain conditions—to arable land, the maps of future land use contain new land areas with wetlands and arable land. Figure 3 shows that the 5000 ad discharge points to large extent are found in arable land or in areas consisting of lakes surrounded by wetlands. Many discharge points are still found in the present-day intake canal to the nuclear power plant and the former bay outside (south-western part of the area, see Fig. 2), which is a wetland on this map. Lake Bolundsfjärden (Fig. 2), which presently is the largest lake in the area, has developed to arable land but contains no discharge points at 5000 ad.

The pattern of the 10 000 ad discharge points on the 10 000 ad land use map is not very different from the corresponding results for 5000 ad (Fig. S2 in Electronic Supplementary Material). The main differences are related to the continued shoreline displacement, with new land areas and lakes forming in the north-east, and succession creating more arable land. Thus, some discharge locations that were found in lakes or wetlands in 5000 ad are in arable land in 10 000 ad. The main impression is that the discharge locations are relatively stable, whereas the land use in the areas where discharge takes place changes. The discharge points calculated for 2000 ad are to larger extent found in the vicinity of the repository, especially in the sea just outside the present coastline (results not shown). This implies somewhat larger changes in calculated discharge locations between the early time steps than later, when most of the area has already changed from sea to land (see Lindborg 2010).

### Analysis of Near-Surface Transport Conditions

The transport modeling carried out in order to study solute discharge and spreading consisted of particle tracking and advection–dispersion simulations based on the surface model developed in MIKE SHE. The starting positions of the particles in the particle tracking simulations were located along the flow paths calculated by the bedrock hydrogeology model. Therefore, differences in discharge locations between the bedrock (ConnectFlow) and surface (MIKE SHE) models would indicate differences in the modeled flow paths from the −40 m level to the surface, and hence could be used to assess the effects of using the more detailed representation of the near-surface domain and the processes therein provided by the surface model. Specifically, such comparisons show whether the same biosphere objects are identified, and also if there are differences in the detailed discharge locations within the objects.

**Fig. 4:**
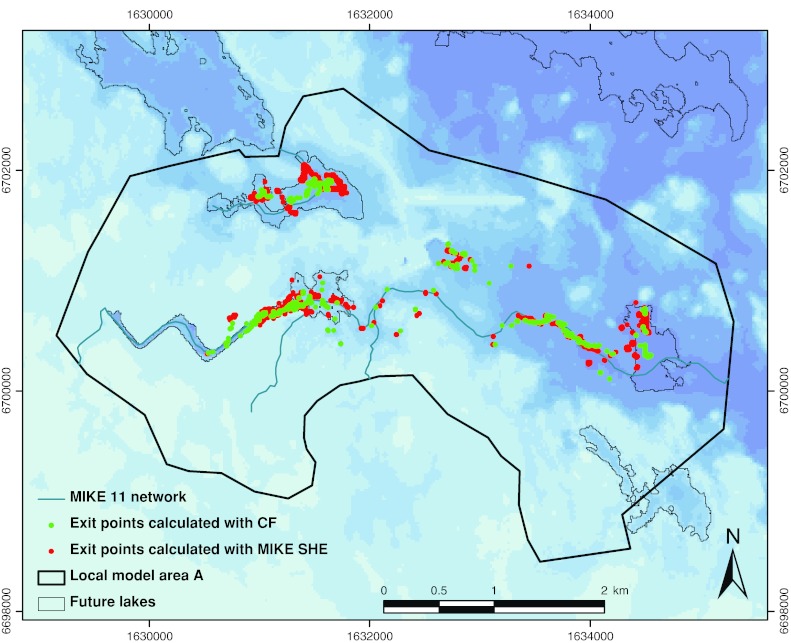
Comparison of discharge points calculated with the surface (MIKE SHE, *red dots*) and bedrock (ConnectFlow, *green dots*) models within one of the local model areas studied in the surface system modeling. Contours of terrestrialized lakes and surface streams are also shown. Reproduced from Bosson et al. (2010)

The results of the particle tracking simulations show that the differences between the results from the bedrock and surface models are very small for the particles going to surface streams (Fig. 4). However, some differences can be observed in the former lake areas. The particles leaving the surface model tend to be more concentrated along the shorelines of the terrestrialized lakes, whereas the particles from the bedrock model appear in the central parts of the lakes. This is probably due to the above-mentioned fact that the bedrock model does not take landscape development into account; since infilling of lakes is not considered, particles will likely to a larger extent continue to discharge in the deep parts of the lakes. The surface model results indicate that the boundaries between the lake areas and their surroundings still have some relevance for groundwater discharge even after the lakes have developed into wetlands and land areas. However, the general impression from the comparison of model results is that the differences in the overall discharge patterns are small.

The MIKE SHE transport modeling considered different source configurations, which in some cases were based on specific scenarios in the safety assessment. For example, one safety assessment scenario focused on transport from the canister positions characterized by the highest groundwater flow rates in the deposition holes and hence by relatively short travel times to the ground surface (SKB 2011). Flow paths from ten of these canisters in the repository were selected and used to obtain source locations for the advection–dispersion modeling with MIKE SHE. Separate simulations were performed for each source/flow path; in the grid cell at an elevation of approximately −40 m along the flow path, a continuous and constant concentration source was set in MIKE SHE.

**Fig. 5:**
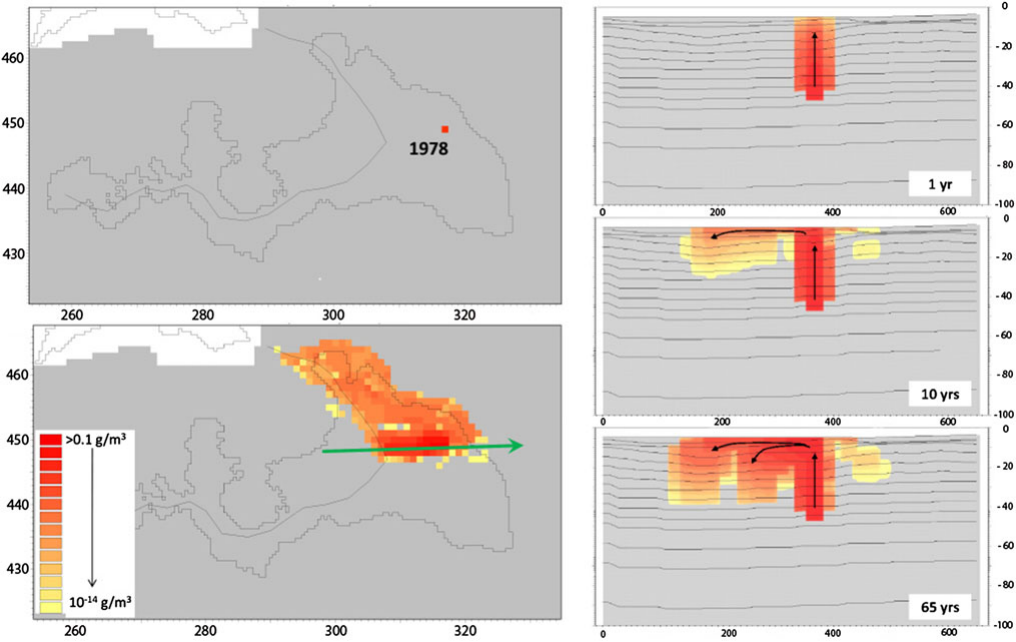
*Left*: Surface plots of a terrestrialized lake and calculated concentration (former lake contour shown); *dark colors* correspond to high concentrations. The *upper figure* shows the location of the source (which is at ca. −40 m), and the *lower figure* the solute concentration in the uppermost layer after 65 years. Note that the length scale is in number of grid cells and should be multiplied by 20 for conversion to meters. *Right*: Concentrations after 1, 10, and 65 years of simulation along the profile indicated by the *green arrow* in the *lower left figure*. Figures reproduced from Bosson et al. (2010)

Figure 5 shows results from one of these simulations; the left part of the figure shows the solute source and the calculated concentration in the uppermost layer of the model, and the right part concentration profiles at different times during the simulation. The strength of the constant source is 1 g/m^3^ but the concentration in the surface layer is very low, except directly above the solute source. The figure shows that the solute is mainly transported directly to the stream (indicated by the line going through the lake area). However, part of the solute mass is spread horizontally over a larger area. The concentration profiles show that the solute first is transported vertically upwards to the surface and when it has reached the surface it spreads in the horizontal direction. The solute spreads both horizontally and vertically from the part of the top layer where it first arrives from below.

The large concentration interval covered in Fig. 5, i.e., from a lowest displayed concentration of 10^−14^ g/m^3^ to the source concentration 1 g/m^3^, is useful to indicate transport directions, but probably exaggerates the area that realistically can be considered affected by transport from the source substantially. To investigate the sensitivity to the selected minimum concentration and perhaps obtain more relevant quantifications of contaminated areas, also a smaller concentration interval with the lowest value at 10^−5^ g/m^3^ was studied (results not shown). The contaminated area is much smaller in this case (ca. 50 m by 200 m, compared to ca. 200 m by 500 m in Fig. 5), but still much larger than the source size (i.e., one cell in the 20 m by 20 m numerical grid).

## Discussion

The uncertainties in the modeled discharge locations need to be assessed as a part of the overall uncertainty assessment of the biosphere modeling and the radionuclide transport and dose calculations. Model results from both bedrock and surface modeling activities are used for this purpose. In particular, the effects of model scale and representation of the fractured medium in the bedrock modeling providing the discharge points have been studied by comparing results from different model variants and cases. These comparisons show that differences can be observed, but these differences are judged not to affect the identification of biosphere objects in the landscape modeling. The same conclusion was reached also when comparing the discharge points calculated in different stochastic realizations, where both fractures and larger deformation zones were handled stochastically, and in parameter sensitivity studies (Joyce et al. 2010).

The sensitivity analysis performed as a part of the bedrock hydrogeology modeling suggests that the description of groundwater recharge and discharge depends on the flow modeling concept, where the Discrete Fracture Network (DFN) approach generates more local flow cells and therefore a larger proportion of discharge points closer to the repository. Due to the Continuous Porous Medium (CPM) representation of the region outside the repository site, the discharge locations may have been unduly dominated by the location of the shoreline. With a DFN (or DFN-based) representation in a larger part of the model volume, the discharge locations would have been more influenced by outcropping deformation zones or fractures than in the present model results.

MIKE SHE simulations with particle releases (particle tracking) or concentration sources (advection–dispersion modeling) at approximately −40 m at locations obtained from flow paths calculated with the ConnectFlow bedrock hydrogeology model showed that near-surface transport is directed more or less vertically up to the regolith, where horizontal spreading takes place. Discharge locations were concentrated to the surface streams and the terrestrialized lakes. In particular, the particle tracking yielded discharge points along the former lake shorelines, rather than in the central parts of the lakes. One reason for this is probably that the relatively low hydraulic conductivities of the lake sediments made the particles move towards the shorelines instead of through the sediments.

The comparison between discharge points obtained from the ConnectFlow (bedrock) modeling and the MIKE SHE (surface/near-surface) modeling showed that the results are similar in terms of the overall discharge patterns and regarding objects receiving particles, whereas there are some differences in the detailed discharge locations. In the areas of the terrestrialized lakes, the discharge points in the MIKE SHE model are to large extent found along the shorelines of the lakes, whereas the particles in the ConnectFlow model tend to appear some distance inside the shorelines. This can most likely be explained by the more detailed representation of the regolith in the surface model, and the fact that changes related to landscape development (including sedimentation and infilling of lakes) are not considered in the ConnectFlow model. For the particles going directly to surface streams, the differences between the results from the two models are very small.

The transport modeling results illustrate the differences between the so-called ‘target area’ where the repository is located (see, e.g., Selroos and Follin 2010) and where horizontal structures (sheet joints) are present in the upper part of the bedrock, and other parts of the future land and lake areas in Forsmark. The sheet joints have a large influence on upward flow and solute transport from the deeper bedrock as well as on downward flow and transport from the surface. They act as drains for water coming both from above and below. Once the potentially radionuclide-bearing groundwater enters a layer with structures of high horizontal conductivity, it is transported horizontally towards the northern part of the model area where discharge occurs.

The results of the advection–dispersion simulations show that solute spreading in some cases leads to relatively large areas with solute in the surface layer, even if the sources are small and relatively close to the surface. In some of the simulations, extensive spreading takes place already in the bedrock, whereas others show large differences between contaminated areas in upper rock and regolith. Hence, one interpretation could be that there is no such thing as a typical pattern of near-surface solute spreading. However, solute transport is generally directed towards the surface water system, which at the modeled time consists of the stream network on the surface.

The MIKE SHE results emphasize the importance of the surface water, which at the considered time consists of the stream network only, for near-surface solute transport in discharge areas. Results similar to those in Fig. 5 were obtained also for several other flow paths. However, in some simulations somewhat different patterns of solute spreading were observed, i.e., both examples of much less spreading in the regolith and cases with more extensive spreading in the bedrock than that observed in Fig. 5 (Lindborg 2010; Bosson et al. 2010). Other modeling cases than those focusing on the selected high-velocity flow paths were also studied. A full account of these simulations is not given here, but it can be noted that most model results indicate more or less vertical transport in upper bedrock and regolith. However, there are also cases where solute injections in areas where flow and transport conditions are affected by the sheet joints in the near-surface rock lead to horizontal transport over larger distances (see discussion in Lindborg 2010 and results presented by Bosson et al. 2010).

## Conclusions

Some variety in the near-surface transport conditions in different parts of the area was found. However, the results also show common features, especially for new land areas at some distance from the planned repository, such that the initial transport from the modeled near-surface sources is mainly vertical and that the highest concentrations are found within relatively small areas and usually directly above the sources. Finally, it is noted that the delimitation of a contaminated area based on advection–dispersion modeling is a matter of definition, since different concentration intervals give different impressions of the degree of spreading and hence of the size of the area affected. This implies that the concentration interval of interest must be specified when assessing the size of contaminated areas, for instance, in connection with regulatory issues.

## Electronic supplementary material

Below is the link to the electronic supplementary material.

Supplementary material 1 (PDF 877 kb)
