# Supplementary material for: Identification and Characterization of Potential Discharge Areas for Radionuclide Transport by Groundwater from a Nuclear Waste Repository in Sweden
Source: Ambio. 2013 Apr 26;42(4):435–46. doi: 10.1007/s13280-013-0395-5 (PMC3636371; doi:10.1007/s13280-013-0395-5)
Supplement: Supplementary file 1 — Supplementary material 1 (PDF 877 kb) [file 13280_2013_395_MOESM1_ESM.pdf]

Electronic Supplementary Material

Identification and Characterization of Potential Discharge Areas for Radionuclide Transport by Groundwater from a Nuclear Waste Repository in Sweden

Sten Berglund, Emma Bosson, Jan-Olof Selroos, Mona Sassner

APPENDIX

Modeling illustrations

Figure S1 below illustrates how the modeling of the hydrologic cycle under present conditions in the Forsmark site description and during the initial temperate period in the safety assessment is divided into a surface-based system and a bedrock-based system. These two systems are modeled with the MIKE SHE and ConnectFlow modeling tools, respectively.

Figure S2 shows discharge points for the 10 000 AD release on a map showing land use at 10 000 AD. The land-use map is produced assuming that all potentially arable land is used for agriculture. Processes leading to infilling of lakes are taken into account.

REFERENCES

Lindborg, T. 2010. Landscape Forsmark – data, methodology and results for SR-Site. Svensk Kärnbränslehantering AB, SKB TR-10-05, Stockholm, Sweden, Report, 252 pp.

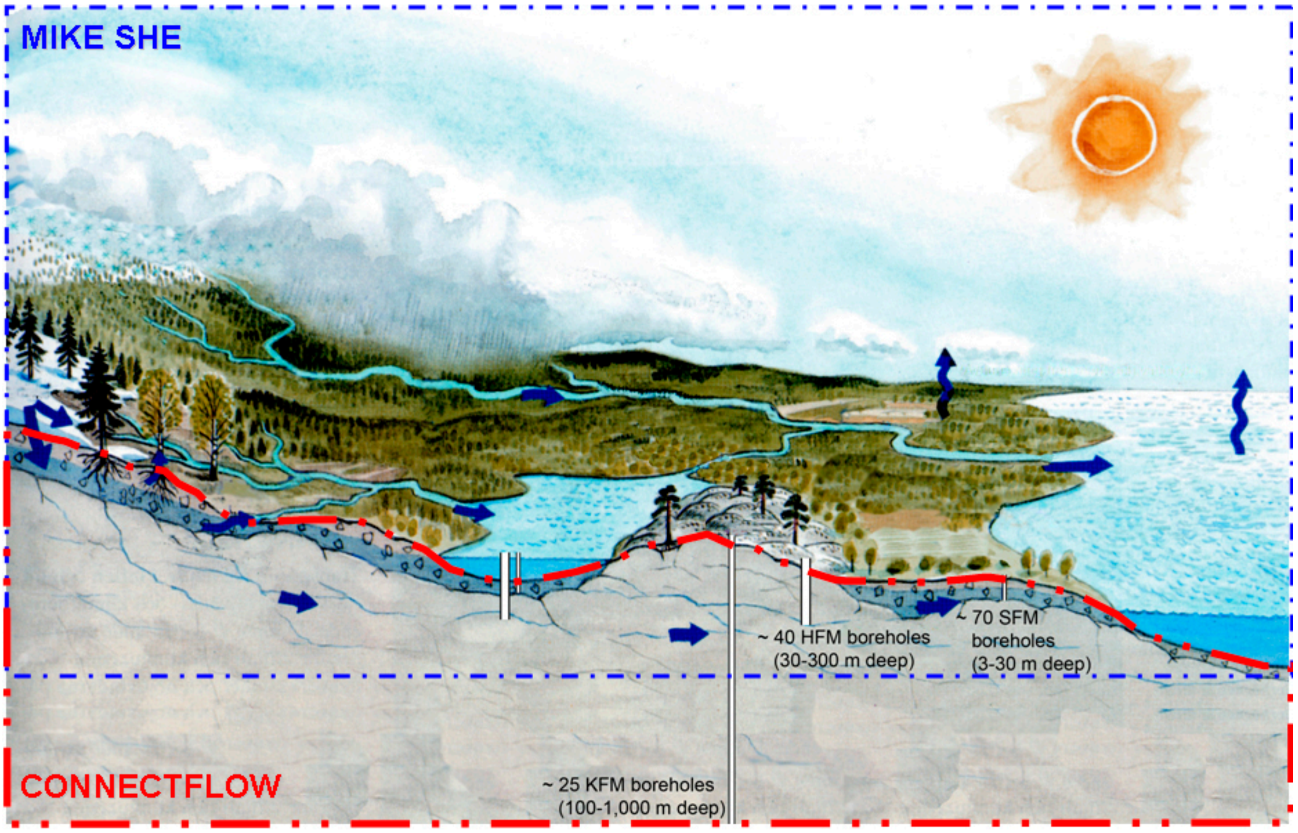

Fig. S1 Modeling of the hydrologic cycle under present conditions in the Forsmark site description and during the initial temperate period is divided into a surface-based system (modeled with the MIKE SHE tool) and a bedrock-based system (modeled with the ConnectFlow tool). SFM, HFM, and KFM denote groundwater monitoring wells in regolith, percussion-drilled bedrock boreholes and core-drilled bedrock boreholes, respectively. Reproduced from Lindborg (2010)

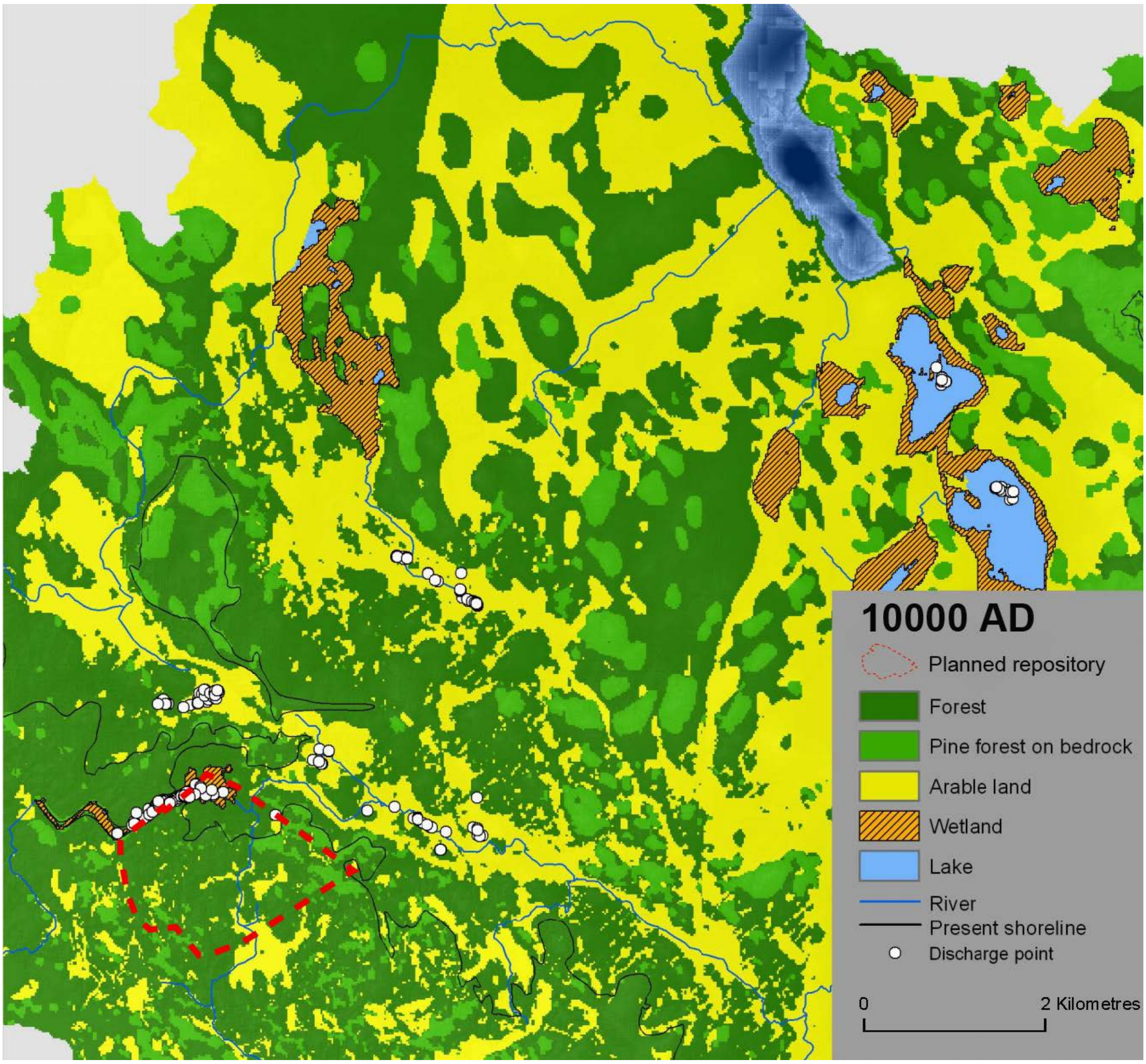

Fig. S2 Discharge points for the 10 000 AD release on a map showing land use at 10 000 AD. The red dotted line indicates the extent of the planned repository. All potentially arable land is assumed to be used for agriculture. Processes leading to infilling of lakes are taken into account. From Lindborg (2010)
